# Effect of formulation parameters and process on the structural properties of concentrated Pickering emulsions

**DOI:** 10.1039/d5ra08955g

**Published:** 2026-01-21

**Authors:** Diego M. Ramos, Mohammad Mahdi Assaf, Véronique Sadtler, Philippe Marchal, Cécile Lemaitre, Tayssir Hamieh, Lazhar Benyahia, Thibault Roques-Carmes

**Affiliations:** a Université de Lorraine, CNRS, LRGP F-54000 Nancy France veronique.sadtler@univ-lorraine.fr thibault.roques-carmes@univ-lorraine.fr; b Faculty of Science and Engineering, Maastricht University P.O. Box 616 6200 MD Maastricht The Netherlands t.hamieh@maastrichtuniversity.nl; c Institut des Molécules et Matériaux du Mans (IMMM), UMR 6283 CNRS – Le Mans Université 1, Avenue Olivier Messiaen 72085 Le Mans cedex 9 France

## Abstract

Concentrated non-conventional anti-Bancroft Pickering direct oil-in-water emulsions stabilized with partially hydrophobic silica particles are addressed. The dispersed-phase fraction of oils is varied between 0.1 to 0.65. A special focus is put on the emulsions formulated at a dispersed-phase fraction of 0.65. The effects of formulation parameters (1 wt% and 4 wt% of silica particles) and emulsification processes (sonicator and rotor–stator shearing devices) on a particle's repartition and organization, and on the resulting rheological behavior of the emulsions are investigated. Emulsions are mainly characterized by droplet size distribution measurements, confocal microscopy images, partitioning of the particles in the emulsion *via* a mass-balance approach, and rheology. The rheological structural properties of the emulsions were probed *via* the study of the dependency of the viscosity on fraction of the dispersed-phase. A modified model of the rheological behavior based on the minimum energy dissipation energy principle, 
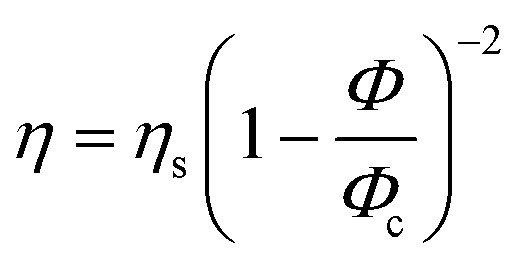
 (Quemada's model), describes fairly well the experimental data. In particular, the two fitting parameters, *η*_s_ and *ϕ*_c_, are sensitive to the repartition of the particles in the emulsions resulting in variation of the stirring process and the amount of silica. We exploit here the 2 parameter-model adjustment which provides a rheological signature of the samples associated to the repartition of the particles inside the system that leads to an evolution of their rheological characteristics. This approach is applied here by playing on the process (rotor–stator and sonication) without changing the physico-chemical parameters. This rheological signature corresponding to the repartition of the particles inside the system is confirmed by other characterization means including confocal laser scanning microscopy and mass-balance approaches.

## Abstract 2

Concentrated non-conventional anti-Bancroft Pickering direct oil-in-water emulsions stabilized with partially hydrophobic silica particles are addressed.

## Graphical abstract

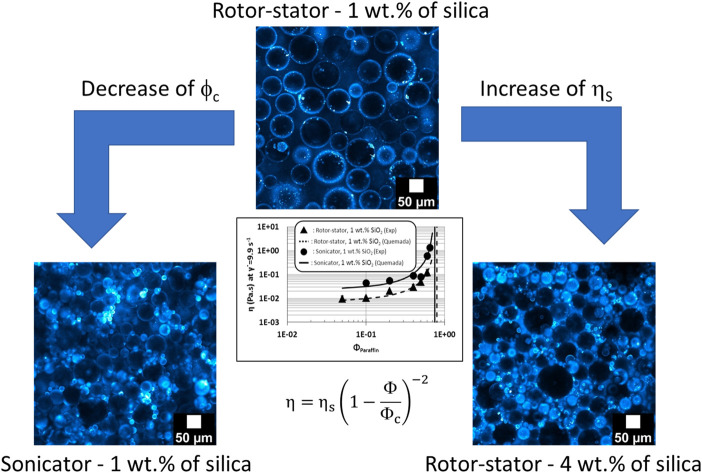

## 1. Introduction

Pickering emulsions are claimed to be very stable against coalescence. This special feature has attracted scientists' and industry's attention. Study and development of new products based on Pickering technology has met recent success^1–3^ or even in development of new biomedical applications.^4,5^ Generally, the type of Pickering emulsion is driven by the contact angle or the hydrophobicity degree of the stabilizing particles.^6^ The Finkle law, similarly to the Bancroft concept for surfactant-stabilized emulsions, states that hydrophobic particles with oil/water/particle contact angle lower than 90° are more appropriate to formulate and stabilize reverse water-in-oil W/O emulsions.^7^ On the opposite, hydrophilic particles (contact angle larger than 90°) are used for direct O/W Pickering emulsions. However, it was recently highlighted that this approach is too simple since some particular particles are able to stabilize both direct and reverse emulsions.^8^ This particular behaviour is mainly attributed to the roughness of the surface of the particles.^9,10^ Recently, this type of trend has been reported with a special type of silica, namely partially hydrophobized HDKH30.^11^ It seems interesting to study further the behaviour of those non-conventional anti-Bancroft direct O/W emulsions stabilized by partially hydrophobic silica particles.

The stabilization mechanism of Pickering emulsions is generally attributed to the irreversible adsorption of the particles at the surface of the droplets. This mechanism is called as pure Pickering stabilization mechanism. In parallel, a network of particles inside the continuous phase can also come into play to stabilize the Pickering emulsions.^12^ Under certain circumstances, the two mechanisms occur simultaneously.^13^ The knowledge of the stabilization mechanism is important since it affects the final macroscopic properties of the final dispersed products.^14^

The organization of phases and particles into Pickering emulsions has been studied for different systems as it defines their rheological behavior and, more particularly, is related to its structural behavior. In other words, organization of the dispersed objects (*i.e.*, droplets and stabilizing particles) plays an important role on definition of their macroscopic properties.^15–17^ A way to probe the organization and interactions of the particles in Pickering emulsions is to follow the evolution of the rheological properties with the fraction of dispersed-phase.^18^ In order to sharpen the comprehension, a rheological model based on the principle of minimum energy dissipation (Quemada's model)^19^ can be applied to the experimental data. This model describes the viscosity of (liquid or solid) suspensions as a function of the fraction of dispersed-phase. It assimilates suspended objects to hard spheres having the same size and neutrally charged in surface (so no interaction with the continuous phase, like hydration). However, droplets in liquid suspensions (*i.e.*, emulsions) behave more like soft spheres, they may be deformed. Furthermore, stabilizing agents (*i.e.*, solid particles) at liquid/liquid interfaces add additional volume to droplets (steric effect)^20^ or they even may produce hydration of droplets surface, increasing also their volume.^21^ Understanding the behaviour of each suspended object in such complex systems to realise predictions on its properties is not that easy. A global view of the complex system seems to be a good approach. This is why a model considering an “effective volume” must be employed when modelling rheological behaviour of Pickering emulsions.^22^ This effective volume takes into account the contributions of each component (continuous phase and dispersed phase) and will be used in the context of this study.

It is then interesting to study concentrated non-conventional anti-Bancroft direct paraffin oil/water emulsions stabilized with partially hydrophobic silica particles. We investigate the effect of formulation parameters (amount of silica) and the emulsification process (type of energy of stirring) on the emulsion's properties (droplet size and stability), particle organization in the continuous phase and at the interface (particle mass-balance and confocal microscopy), and the rheological properties. Two stirrers delivering different power are employed, *i.e.*, ultrasonic probe (∼10^9^ W m^−3^) and rotor–stator mixer (∼10^7^ W m^−3^), to emulsify Pickering systems ranging from 0.10 to 0.65 of paraffin oil fraction and stabilized with 1 wt% of silica particles. Another series was carried out with the rotor–stator for emulsions stabilized with 4 wt% of silica particles. In particular, we discuss the structural properties of the emulsions by rheological modelling and the repartition of the particles at the interfaces and in the continuous phase in relation with the process and the silica content.^23^

## 2. Materials and methods

### 2.1. Chemicals

Paraffin oil was obtained from Fisher Scientific and was general-purpose grade. The aqueous phases contained 2% of NaCl. The NaCl was provided by Sigma-Aldrich and had a purity of 99.5%. The stabilizing particles were dried silica particles partially hydrophobized. They were silica HDK H30 received from Wacker Chemie. Previous analysis indicated that the silica/paraffin oil/water contact angle was of the order of 122°. This high contact angle value corresponded to hydrophobic silica particles. The diameter of the dried native particle was measured as 20 nm. We were aware that the NaCl concentration affects drastically the double layer range of silica particles. Hence, we conducted all the experiments at constant values of NaCl concentration of 2 wt%. It was decided to work at constant but large NaCl content in order to efficiently screen the double layer range and surface charge of silica particles. The fluorescent dye used here was Rhodamine B (Sigma-Aldrich).

### 2.2. Pickering emulsions preparation

Preparation of the suspensions of silica particles and the direct O/W emulsions is described in this section. Firstly, the dispersion of the particles in water is described. Then, the dispersion and homogenization of an oily phase into the silica suspension by two different processes are presented. Each process was characterized by the emulsification tool, *i.e.*, a rotor–stator mixer or an ultrasonic probe.

First, dispersion of silica particles was formulated. They contained 1 wt% or 4 wt% of silica content relatively to water volume. A first agitation step with a magnetic stirrer was conducted during 48 hours in order to wet the hydrophobic particles in the aqueous medium. Then, a sonication step was performed with a 550 Fisher sonic probe during 20 min at a power of 20 W. Each ultrasound pulsation lasted 2 s followed by 2 s with no power. An ice bath was used to avoid the rise of temperature during the sonication step. The particles size of the silica aggregates after suspension preparation were measured *via* dynamic light scattering (DLS) with a Malvern high performance particle sizer (HPPS) instrument. The size of the particles was recorded as 402 ± 293 nm for 4 wt% of silica while the diameter became equal to 197 ± 84 nm for 1 wt% of silica.

For the emulsions, batches of 70 mL were prepared. The fraction of paraffin oil was varied between 0.1 and 0.65, respectively, to the volume of the emulsion. Each formulation was subjected to both emulsification processes (*i.e.*, rotor–stator mixer or ultrasonic probe). Dilution tests were utilized to confirm the nature of the O/W emulsions.^24^

In the following, the name “rotor–stator emulsions” was used to speak about the emulsions prepared with a rotor–stator stirring device. The emulsions were prepared in a semi-batch mode. The paraffin oil was contained in a syringe. In parallel, the silica suspension was inserted in the beaker. An initial volume of 1 mL of paraffin oil was introduced dropwise, using the syringe, into the silica aqueous suspension. Immediately after contact, the rotor–stator was started up at 13 500 rpm for 20 s (UltraTurrax® DI 25 Basic fitted with an S 25 N-10G rod from IKA-Werke GmbH & CO). A further 1.0 mL of oil was then added to the emulsion. This procedure was repeated until a volume of paraffin oil was sufficient to achieve the desired volume fraction of dispersed phase (from 0.1 to 0.65). At the end of the addition, the emulsion was kept under stirring for 5 minutes by the means of the UltraTurrax® at 13 500 rpm.

The emulsions produced by the ultrasonic emulsification process will hereinafter be referred to as “sonicator emulsions”. They were prepared by first dispersing the oil phase in semi-batch mode. This dispersion process followed the same protocol as for the preparation of rotor–stator emulsions. Next, the homogenization and emulsification process were carried out using a 550 Fisher sonicator. Power was set at 120 W for 2 s ultrasonic pulses. Total ultrasonic exposure time was set at 10 minutes. To ensure complete incorporation of the oil and recirculation flow, the emulsions were stirred once more with the rotor–stator for 10 minutes. This was followed by 10 minutes of ultrasonic homogenization. An ice bath was used to avoid the rise of temperature during each sonication steps.

Note that the sonicated emulsions were only prepared with 1 wt% of silica. The systems containing 4 wt% of silica were too viscous, mainly at high dispersed-phase fractions of 0.60–0.65, to be efficiently prepared with the sonicator process.

### 2.3. Characterization

Four main characterizations of emulsions were proposed: droplet size distribution, microscopical characterization, an experimental material balance, and viscosity behavior.

A MasterSizer 2000® was used to probe the droplet size distribution of the emulsions. The apparatus was based on static light scattering technique (SLS) coupled with the Fraunhoffer diffraction model. All measurements were performed by triplicate.

Confocal microscopy had been used to characterize the repartition of the particles inside the emulsions. This technique enabled us to visualize the organization of silica particles in the continuous phase and on the surface of droplets. A ZEISS International LSM 800 confocal microscope was employed. A Magnification of ×25 was used. Only the particles were labelled with rhodamine B. A rhodamine B concentration of 10 ppm in water was used and mixed with the suspension of silica particles prior to emulsification.

With the purpose of quantifying the partitioning of the particles into emulsions, an experimental material balance was carried on following a procedure previously described.^25^ It consisted in separating the continuous phase from the dispersed phase of the emulsions without breaking the emulsion. In other words, centrifugation of the emulsions (Allegra X-22 centrifuge, Beckman–Coulter) at 3000 rpm for a duration of 30 min was performed. At the end of the centrifugation process, the continuous phase was withdrawn from the system in order to estimate the mass of silica remaining in the continuous phase. For that purpose, a known volume of the continuous phase was heated up to 180 °C to remove the water. The mass of the final white powder corresponding to the silica initially present in the continuous phase was measured and was denoted as *m*_Silica, Continuous_. In parallel, the total mass of silica in the emulsion was denoted as *m*_Silica, Total_, the particles density as *ρ*_Silica_, the size of aggregates as *V*_Silica aggregate_, and *A*_Interfacial_ was the interfacial area.

The quantity of particles adsorbed at the interfaces (Γ) was calculated using:1
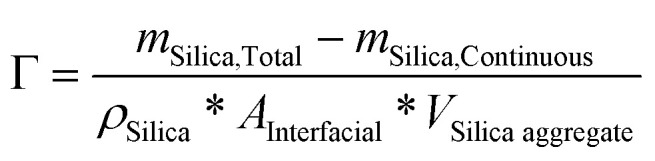


Based on our previous studies, the silica aggregates were assimilated to spheres and their size to 10 µm for rotor–stator emulsions and 400 nm for the sonicator ones.^26^ Every experiment was repeated three times. For each dispersed-phase fraction tested, 3 separate emulsions were formulated.

In order to confirm that no droplet breakage took place, control experiments were conducted to validate this assumption and confirm that the emulsions remain stable after such a gravity stress. To this extent, after phase separation (centrifugation of the emulsions at 3000 rpm for 30 min), the cream was recovered and stored in a dark room at a constant temperature of 20 °C. After 1 day, the cream was redispersed in a silica-free continuous phase (water containing 2% of NaCl). The droplet size distributions of these emulsions were measured and compared with those of fresh emulsions. The results are reported in Fig. S1 in SI. No substantial variation of the droplet size distributions was observed between fresh emulsions and redispersed creams. These results highlight that the emulsions remain stable after such a gravity stress and also the absence of droplet breakage even after centrifugation.

**Fig. 1:**
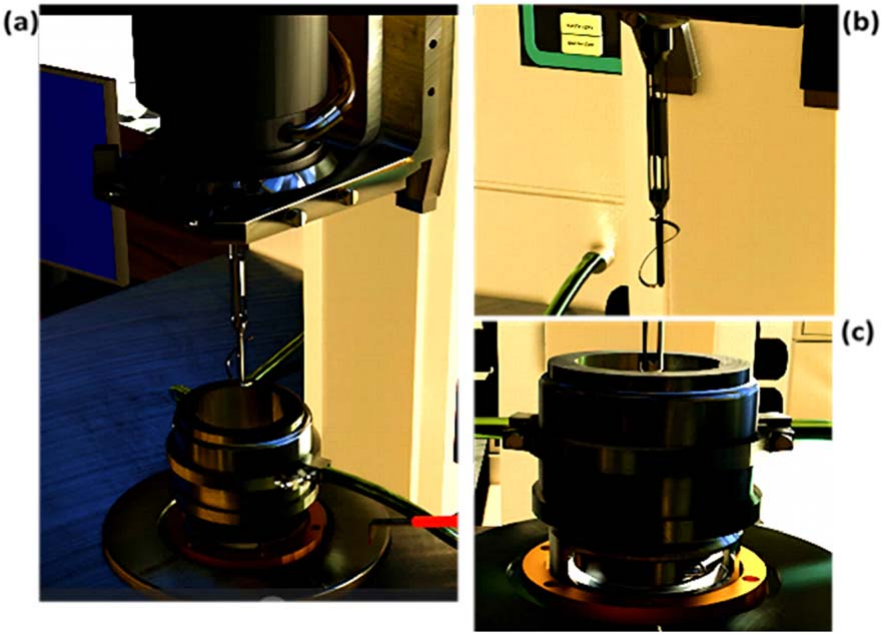
Presentation of (a) the rheo-reactor system, (b) the static stirring device (torque captor), and (c) the cylindrical tank (shear generator).

For rheological measurements, it is important to avoid the creaming of the emulsions which could introduce an artifact during the measurements. Axial mixing of Pickering emulsions controls this behavior by gently continuously mixing the system.^27^ A rheo-reactor previously developed in our laboratory was well adapted to avoid the creaming (Fig. 1a). The rheological behavior of emulsions was then characterized with the rheo-reactor. The measuring cell consists of a cylindrical tank 6.8 cm high with a diameter of 5.5 cm (Fig. 1c). The pitch of the helical ribbon was 4.2 cm for a diameter of 4.5 cm (Fig. 1b). All tests were carried out on an ARES type-imposed strain rheometer (TA Instruments) at 20 °C, the temperature being controlled by a thermostatic bath. The gap between the bottom of the tank and the end of the tape was set at 4 mm. For this special geometry, the value of the stress constant was 0.90988 Pa g^−1^ cm^−1^ and that of the strain was 2.749 rad^−1^. The shear rate was imposed *via* the rotation of the tank and the torque was measured on the axis of the helical ribbon which is static.

The rheological tests were performed following the following protocol. Firstly, the emulsion was gently stirred, generating a vortex, to obtain a homogeneous system and a representative sample. Then, the emulsion was introduced inside the rheo-reactor and the helical ribbon was lowered. Steady-state flow tests were performed between 100 s^−1^ and 1 s^−1^ in logarithmic mode. Measurements were not performed below 1 s^−1^ in order to maintain sufficient agitation during the tests and to avoid creaming of emulsion droplets and sedimentation of silica particles. For each imposed shear rate, the pre-measurement delay and the measurement time were set to 30 s each. Each flow curve was performed three times per sample. For each dispersed-phase fraction tested, 3 separate emulsions were formulated. The flow curves thus obtained (viscosity as a function of shear rate) were then used for samples of dispersed-phase fractions between 0.1 and 0.65.

## 3. Results and discussion

### 3.1. Droplets size

**Fig. 2:**
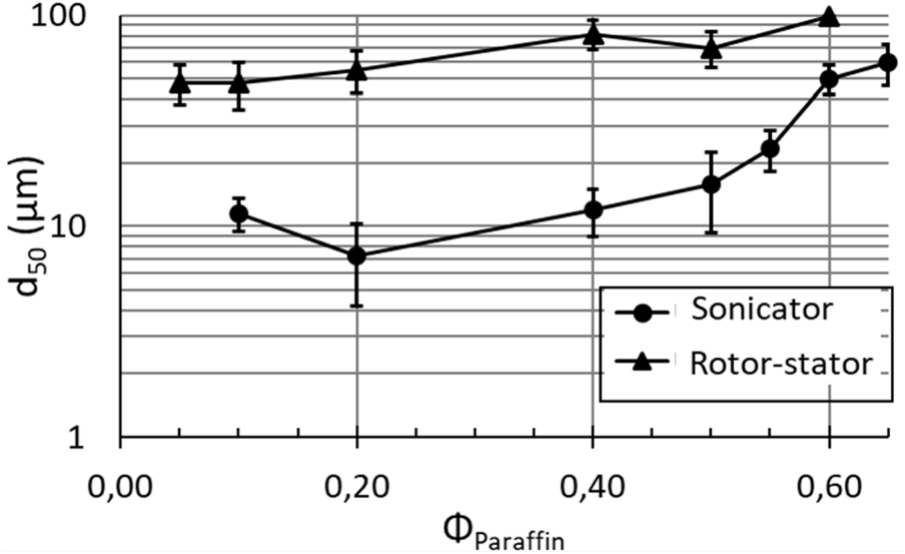
Evolution of the average droplet size (*d*_50_) of droplets in rotor–stator and sonicator emulsions, prepared with 1% wt of particles, as a function of the content of paraffin oil dispersed-phase (*ϕ*_Paraffin_). The lines are drawn to guide the eyes.

Fig. 2 shows the variation of the average droplet size of emulsions with the fraction of paraffin dispersed-phase. Rotor–stator (black triangles) and sonicator (black circles) emulsions were stabilized with 1 wt% of silica particles in water. As expected, for all the preparations, average droplet sizes of sonicator emulsions (10–60 µm) were smaller than those of rotor–stator emulsions (50–100 µm). This gap is due to difference between energy delivered by the sonicator (∼10^9^ W m^−3^) and the rotor–stator (∼10^7^ W m^−3^) during emulsification processes. The sonicator device, which is the most energetic, produces the largest interfacial area leading to the smallest droplets which are efficiently stabilized by the particles.

Also, average droplet size increases with the paraffin oil fraction in all the emulsion systems (Fig. 2). Eqn (2) shows estimation of average droplet diameter (*d*) of Pickering emulsions from the volume of dispersed phase (*V*_d_) and the quantity of particles (*m*_p_), besides other parameters as the size of stabilizing particles (*d*_p_), their density (*ρ*_p_) and the coverage fraction (by particles) of droplets interfaces (*C*).^28^ It is called the “limited-coalescence model” and reads as:2
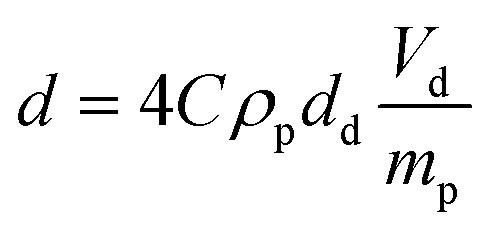


On the one hand, droplet size increases with the paraffin oil fraction. On the other hand, since the silica content is referred to the continuous phase of emulsions, the quantity of particles into emulsions diminishes as the fraction of paraffin oil increases. Emulsions prepared with suspensions of 1 wt% of silica particles and 0.1 of paraffin fraction contain more silica (0.9 wt% referred to the emulsion volume) than those with 0.65 (0.35 wt% referred to the emulsion volume). In consequence (and from eqn (2)), it is expected that average droplet size of emulsions increases with the fraction of paraffin oil. The experimental evolution of the average droplet diameter follows the trend predicted by the “limited-coalescence model” (eqn (2)).

It is also interesting to investigate the polydispersity of the emulsions. To discuss this aspect, the span values obtained from the droplet size distribution are reported in the Table 1.

**Table 1:** Span values obtained from the droplet size distribution

Fraction of paraffin oil dispersed-phase (*ϕ*_paraffin_)	Rotor–stator emulsions 1 wt% silica	Sonicator emulsions 1 wt% silica	Rotor–stator emulsions 4 wt% silica
0.05	1.336	—	—
0.1	1.353	1.846	—
0.2	1.519	1.683	1.580
0.4	1.047	2.675	—
0.5	1.438	1.504	1.495
0.55	—	1.513	—
0.6	1.538	1.971	1.735
0.65	—	1.564	—

The span values range between 1.047 and 1.971 for all the system. This indicates that the emulsions are mainly monodisperse. At 1 wt% of silica, the span values are lower for the rotor–stator emulsions in comparison to that prepared with the sonicator. The high energy of the sonicator reduces the size of the droplets but increases slightly the span values. For the rotor–stator emulsions, the increase of the silica content from 1 wt% to 4 wt% does not significantly affect the span value. As a general tendency, the span value in the presence of 4 wt% of silica lies between the span values of the 2 emulsions with 1 wt% of silica prepared with the 2 shearing devices.

**Fig. 3:**
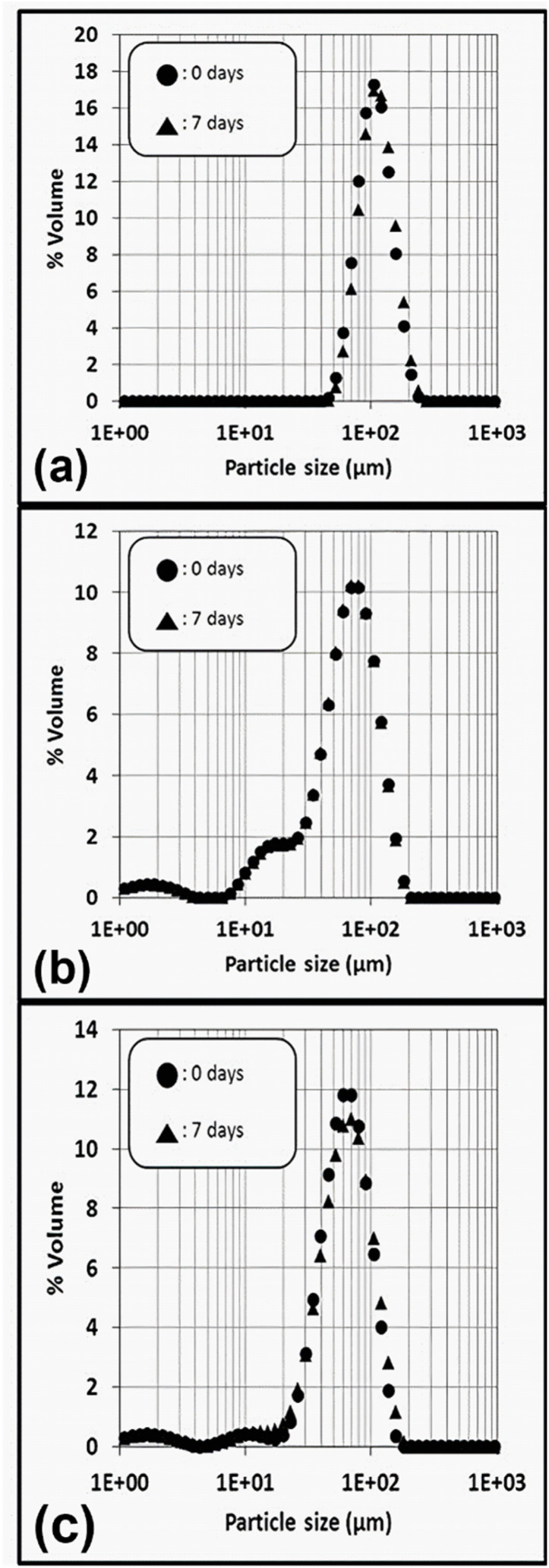
Evolution through 1 week of droplet size distributions of the Pickering emulsions with paraffin oil fraction of 0.65. Emulsions with 1 wt% of silica particles, prepared with (a) the rotor–stator or (b) the sonicator device. (c) Emulsions with 4 wt% of silica particles prepared with the rotor–stator device. “0 days” represents the droplet size distribution just after preparation. “7 days” corresponds to the droplet size distribution after 1 week of storage.

It is also relevant to specifically study the emulsions at the higher dispersed-phase fraction of 0.65. For this purpose, an additional emulsion prepared with rotor–stator and containing 4 wt% of particles was prepared. Fig. 3 displays the droplet size distribution of the rotor–stator emulsions prepared with 1 and 4 wt% of silica as well as the sonicator emulsion prepared at 1 wt% of silica (“0 day”). All the emulsions contain a dispersed oil-phase fraction of 0.65.

The top of the main peak is identical for the 3 emulsions at around 90 µm. However, smaller population of droplets, of around 10–20 µm, appears by increasing the silica content (Fig. 3c) and shifting the stirrer to high energy sonication (Fig. 3b).

### 3.2. Confocal microscopy

**Fig. 4:**
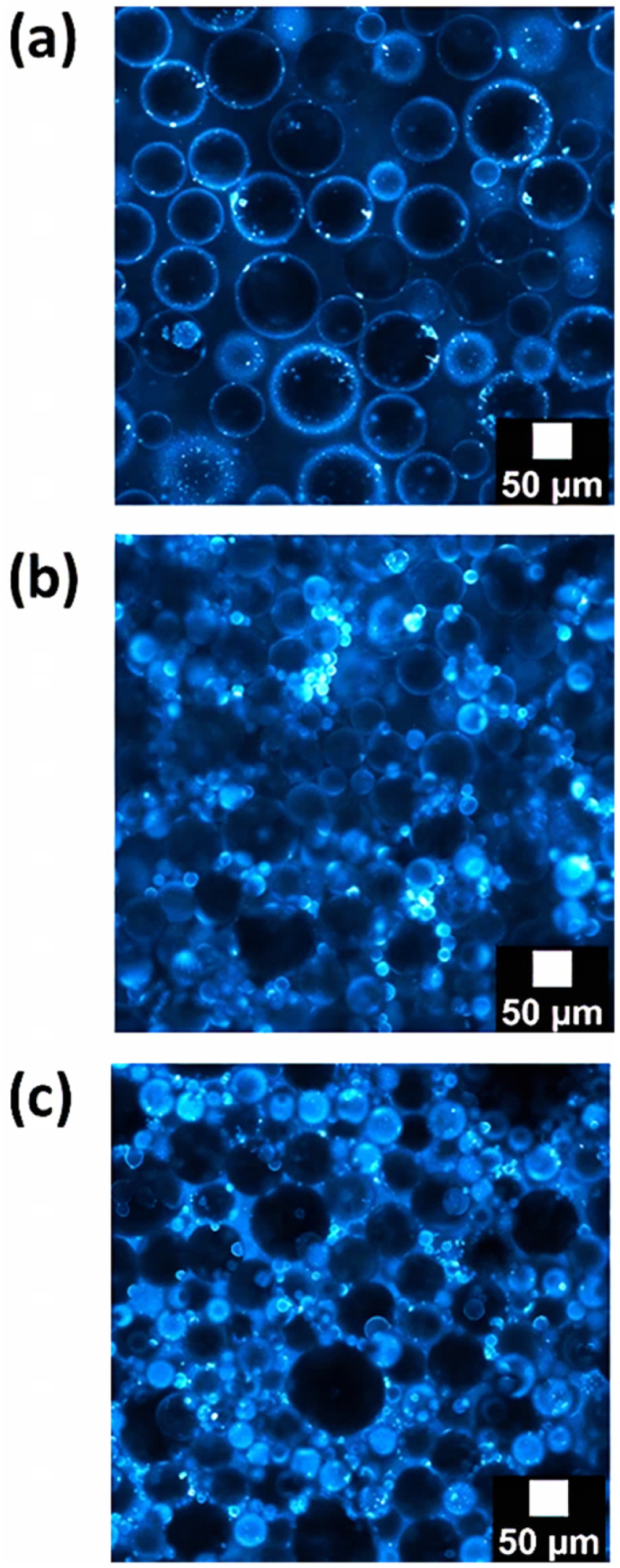
Confocal microscopy images from emulsions with 1 wt% of silica particles prepared with (a) the rotor–stator device or (b) the sonicator. (c) Image of an emulsion prepared with 4 wt% of particles and the rotor–stator. Fraction of paraffin oil was 0.65 for all the systems. Silica particles are represented in blue.

Fig. 4a and b display confocal microscopy images of emulsions with the highest paraffin oil fraction (0.65) and with 1 wt% of silica particles, prepared with the rotor–stator or the sonicator devices, respectively. The silica particles are represented in blue. Both images show that all the droplets are surrounded by a bluish corona, meaning the stabilization of interfaces by the presence of silica particles. Also, they confirm that average droplets size of rotor–stator emulsions, at this paraffin fraction, is bigger than the one of sonicator emulsions. The smallest droplets size of rotor–stator emulsions, at this paraffin fraction, is bigger than the one of sonicator emulsions. On another note, the smallest droplets (sonicator, Fig. 4b) occupy the whole visual field while some droplet-free regions (in the continuous phase) are present between the biggest droplets (rotor–stator, Fig. 4a). Moreover, the particles at the interfaces of the rotor–stator emulsion are bigger than those at the interfaces of the sonicator one. Also, from the visual texture of the image, we know that some particles remain in the continuous phase of the rotor–stator emulsion.

Fig. 4c displays the image of a rotor–stator emulsion prepared with 4 wt% of particles at a dispersed-phase fraction of 0.65. The most striking feature is the presence of particles in the continuous phase. More precisely, a dense network of particles linking the droplets can be detected. A network of particles was also observed at 1 wt% of silica for sonicator emulsion (Fig. 4b) but the network was less dense.

### 3.3. Surface coverage of particles

**Fig. 5:**
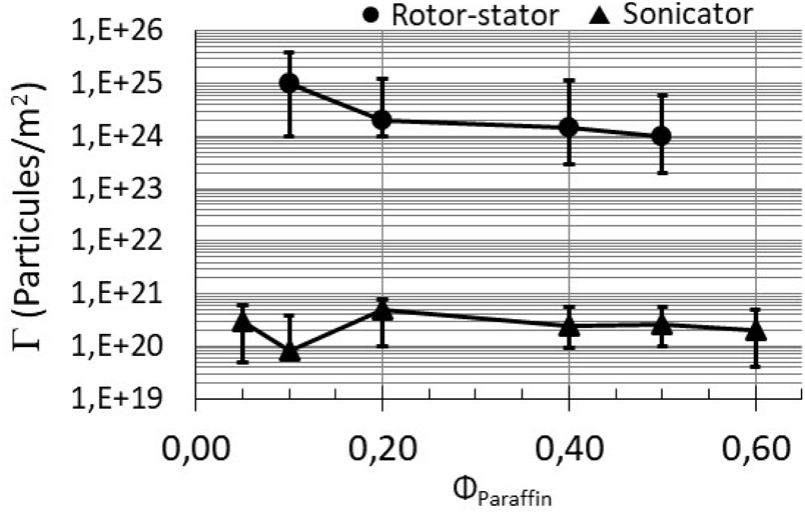
Evolution of the interfacial concentration of silica particles (Γ) while increasing the paraffin oil dispersed-phase content (*ϕ*_Paraffin_) into emulsion. Rotor–stator and sonicator emulsions were stabilized with 1 wt% of silica particles. The lines are drawn to guide the eyes.

In order to quantify the repartition of the particles between the bulk and the interface, a mass-balance approach was used for the particles. We put on emphasize on the amount of particles adsorbed at the droplet interfaces (Γ). The evolution of the interfacial concentration of silica particles (Γ) with the paraffin oil dispersed-phase content (dispersed-phase fraction *ϕ*_Paraffin_) for rotor–stator and sonicator emulsions stabilized with 1 wt% of silica is given in Fig. 5.

Number of particles adsorbed at the interfaces of sonicator emulsions was substantially larger than that of rotor–stator emulsions (about 10 000 times higher). Slight reduction of Γ is observed with the sonicator while it is not substantially affected by the paraffin content with the rotor–stator. The silica adsorbed amount is reduced as the interfacial area increases (sonicator produces the smallest droplets at a same oil fraction). Energy from the sonicator generates the biggest interfacial area and promotes also breakage and diffusion of silica aggregates from the aqueous phase to the interfaces.

In terms of particles repartition, it is interesting to focus on the data at a dispersed-phase fraction of 0.65. Recall that network of particles in the continuous phase was recorded with the sonicator emulsion. Conversely, only a weak amount of particles remained in the continuous phase for rotor–stator emulsion. This indicates that large aggregates of silica are adsorbed onto the droplets with rotor–stator emulsion. On the opposite, smaller particles adsorbed onto the droplets in parallel to a network of small particles in the continuous phase are expected with sonicator emulsion. The interfacial concentration of silica Γ for particle concentration of 4 wt% at a dispersed phase fraction of 0.65 leads to a value of Γ of 10 (ref. 21) particles per m^2^. The value is slightly larger than that obtained with 1 wt% of silica. This data indicates that the additional particles introduced in the emulsions mainly remain in the continuous phase to produce the dense network of particles bridging the droplets.

### 3.4. Stability

It was previously demonstrated that emulsions formulated with 1 wt% of silica and a dispersed-phase fraction of 0.5 remain stable during 60 days regardless of the process used.^26^ In addition, the rotor–stator emulsion was still stable after 90 days.^3^ The stability of the sonicator emulsion was not evaluated after 60 days. It was also previously explained in this study that droplet size of emulsions increased with the paraffin oil fraction not only because the volume of oil was rising but also because quantity of silica particles to stabilize interfaces was diminishing. It is important to figure out if direct emulsions obtained at the highest fraction of paraffin oil (0.65) were stable over time, even if stabilized with particles of partially hydrophobic silica.

Fig. 3 displays evolution of droplet size distribution during 1 week (“7 days”) of emulsions prepared with the maximal fraction of paraffin oil (0.65) for rotor–stator emulsions at 1 and 4 wt% of silica and sonicator emulsion prepared at 1 wt% of silica.

The distributions correspond fairly well to images obtained by confocal microscopy (Fig. 4). At that fraction of paraffin oil, the silica content of a 1 wt% suspension becomes 0.35 wt% when referring to the whole emulsion volume. It becomes 1.4 wt% for a suspension of 4 wt%. Emulsions prepared with the highest silica content and the lowest energetic process (rotor–stator, ∼10^7^ W m^−3^) were stable during that period of time. Interestingly, emulsions stabilized by the lowest quantity of partially hydrophobic silica (and with the highest paraffin content) were also stable over 7 days, independently of the energy delivered by the stirrer (rotor–stator or sonicator) to emulsify the system. No variation of the droplet size distribution can be highlighted during one week regardless of the process and formulation such as silica content. Note also that no clear variation of the macroscopic aspect of the emulsions could be detected after 3–4 weeks but the droplet size distribution was not evaluated. These results highlight that the 2 modes of stabilization of the emulsions are efficient in our systems. For rotor–stator emulsions at 1 wt% of silica, the adsorbed particles at the O/W interfaces successively stabilized the droplets. This can be viewed as a pure-Pickering stabilization mechanism. For the 2 other emulsions, the network of particles in the continuous phase coupled to the adsorbed particles on the droplets are the main mechanisms of stabilization.

### 3.5. Rheological behavior and structural properties

The rheological behavior of the emulsions was studied through flow curves, *i.e.*, viscosity as a function of the shear rate, for dispersed-phase fractions ranging from 0.1 to 0.65 (Fig. S2–S4 of the SI). In parallel, successive applications of loading (increasing stress) and unloading (decreasing stress) cycles were performed to test the possible restructuration of the emulsions under flow. A time duration of 30 s was imposed between each stress rise or drop to erase short time thixotropy effect. Loading and unloading curves were superposed which reveals the absence of alteration of the emulsion structure during the rheological measurements (figures not shown). This was expected based on the range of shear rates between 1 and 100 s^−1^ applied in the rheometer. Actually, in order that the shearing by the rheometer alters the emulsion structure, the energy provided by the rheometer has to be larger than the energy dissipated by the emulsification process. This is not the case here since the emulsions were prepared by the means of sonication and rotor–stator shearing device. The viscosity measured at a shear rate of 9.9 s^−1^ was extracted from each flow curve. It becomes then possible to plot the evolution of the viscosity as a function of the dispersed-phase fraction. This is the first step in the structural analysis of the data.

**Fig. 6:**
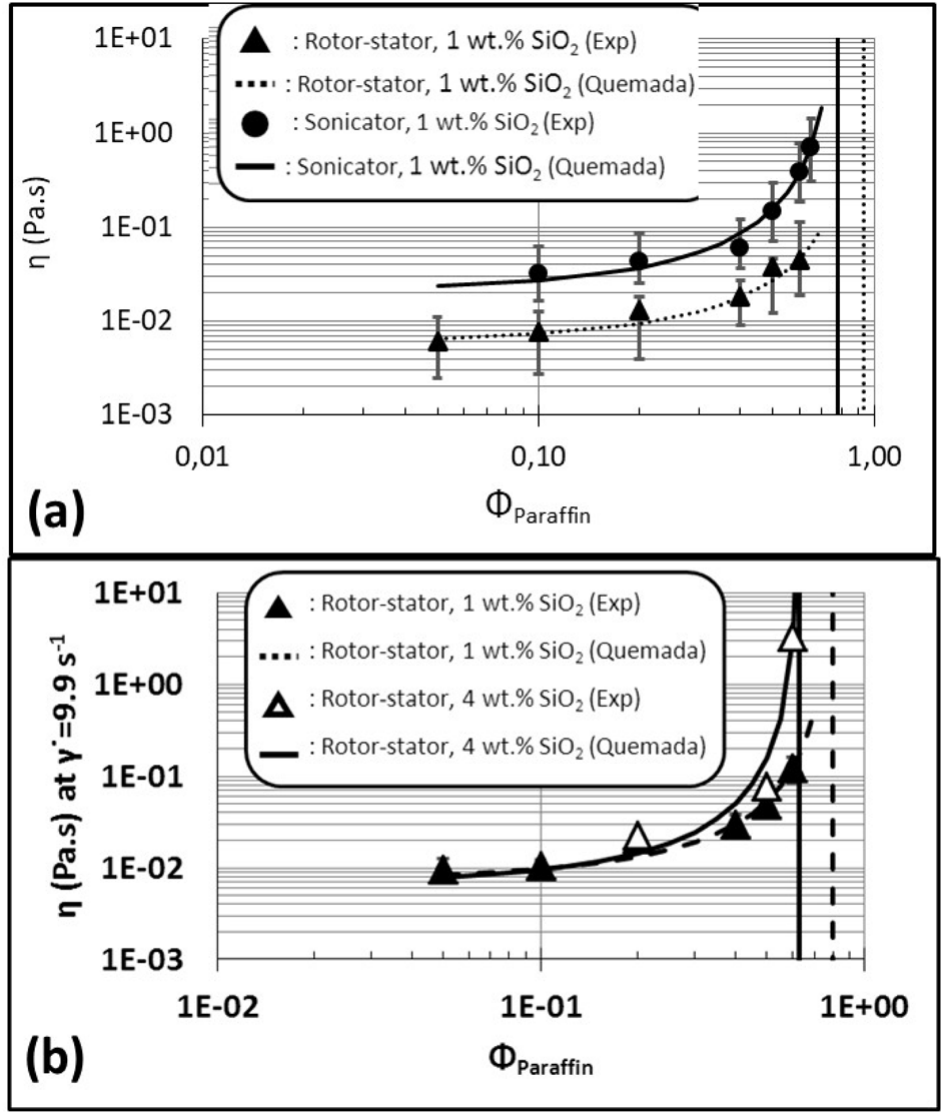
Evolution of the viscosity (*η*) as a function of dispersed-phase paraffin fraction (*ϕ*_Paraffin_) of (a) rotor–stator and sonicator emulsions prepared with 1 wt% of silica particles, (b) rotor–stator emulsions prepared with 1 or 4 wt% of silica particles. The points correspond to experimental data (“Exp”). The lines represent the fit of the data with 
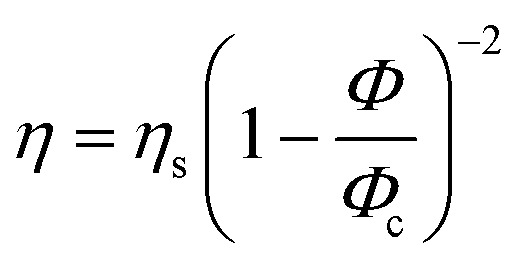
 where *η*_s_ and *ϕ*_c_ are the fitting parameters (“Quemada”).

In a first step, the impact of the process on the evolution of the viscosity with the dispersed-phase fraction is evaluated. Fig. 6a shows the evolution of the viscosity (at a shear rate of 9.9 s^−1^) of emulsions prepared with rotor–stator (black triangles) or sonicator (black circles) and stabilized with 1 wt% of silica particles, as a function of their fraction of paraffin oil. As expected, viscosity of Pickering emulsions increases with the fraction of dispersed-phase. A slight increase of viscosity with the paraffin fraction takes place up to around 0.50 followed by a sharp increase above this fraction. This kind of trend was already reported in the literature.^18,22^ Moreover, it is important to notice that viscosity of rotor–stator emulsions is always lower than the one of sonicator emulsions. This behavior is consistent with the difference of energy delivered to the system by each stirrer in the emulsification process. As expected, energy provided by the sonicator (∼10^9^ W m^−3^) is enough to generate a bigger interfacial area than the one produced by the rotor–stator (∼10^7^ W m^−3^) due to the apparition of smaller population of droplets of around 10–20 µm. The quantity of droplets into sonicator emulsions is consequently higher than that of rotor–stator emulsions. So, more steric interactions among dispersed objects become possible. Sonicator emulsions are then the most viscous at a defined fraction of dispersed phase.

In a second step, the effect of the amount of silica is discussed. Fig. 6b displays the evolution of viscosity of rotor–stator emulsions prepared with two different contents of silica, 1 wt% (black triangles) or 4 wt% (white triangles). In both cases, viscosity of Pickering emulsions increases with the fraction of dispersed phase, as expected. For a same quantity of energy delivered to the system (same emulsification process), increasing the fraction of paraffin oil allows production of a larger interfacial area. So, the quantity of droplets and also of interactions in between increases, rising up viscosity of the emulsion. Also, at the highest fractions, emulsions prepared with the highest content of particles (4 wt%) are the most viscous (3.29 Pa s with 4 wt% *vs.* 0.12 Pa s with 1 wt%). Average droplets sizes of rotor–stator emulsions at the highest paraffin oil fraction are about 90 µm whether stabilized by 1 wt% or 4 wt% of silica particles (Fig. 3a and c, respectively). Consequently, the droplets occupy the same volume regardless of the silica content. However, particles excess in emulsions with 4 wt% of silica (in addition to the apparition of smaller population of droplets of around 10–20 µm) help droplets to fulfill the emulsions volume and so, reach firstly the maximal fraction of dispersed paraffin oil. This aspect is visually confirmed in Fig. 4c where a network of droplets is linked *via* the silica particles (bluish background) in between. Conversely, for the 1 wt% silica emulsion, the droplets are in direct contact (mostly dark background and not bluish in Fig. 4a).

In a second step, a rheological model can be used to interpret the experimental data. One of the best-established expressions to describe the variation in viscosity *η* of a suspension with its volume fraction *ϕ* is:^19,29–31^3
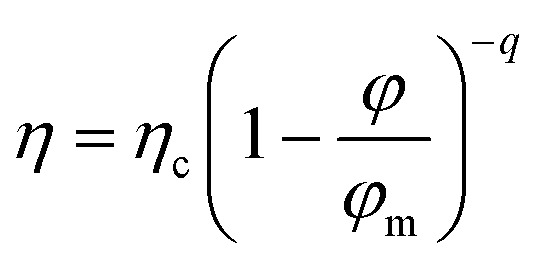


This expression can be established on experimental, phenomenological or theoretical grounds,^19,20^ such as the principle of minimum energy dissipation,^19^ with *q* = 2 in this case (Quemada's model), leading to:4
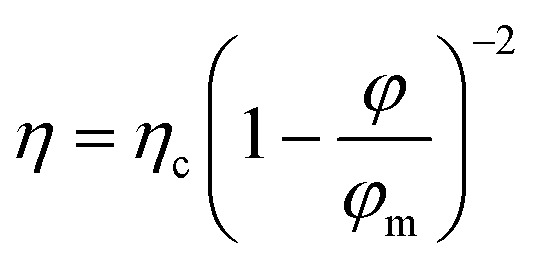
where *η*_c_ is the viscosity of the continuous phase, *q* an exponent and *ϕ*_m_ the volume fraction at which the viscosity diverges. This divergence is akin to a glass or jamming transition associated with the loss of particle mobility at high concentrations, for which the free volume of particles becomes too small to allow suspension flow. For a monodisperse suspension of hard spheres, *ϕ*_m_ is typically not far from the random close packing fraction *ϕ*_rcp_ = 0.637.

Quemada's model was initially developed for hard sphere suspensions. Here, we use the version of the model which was extended to polydisperse, non-spherical, and soft particles.^32,33^ In addition, attractive or repulsive interparticular potentials between particles can be also taken into account into the model through an effective dispersed volume fraction.

Eqn (4) is appropriate for suspensions of monodisperse hard spheres. In the case of suspensions of polydisperse soft particles interacting through an interaction potential (surface electric charges, ionic double layers, polymers or particles adsorbed at interfaces), the volume fraction *ϕ* must be replaced by an effective volume fraction *ϕ*_eff_ = *αϕ*, with *α* ≥ 1, to account for the extent of the interactions potentials and their volume of influence. In this study, the excess volume corresponds to the additional space occupied by silica particles adsorbed on the surface of oil droplets (see Fig. 4b and c). Furthermore, the polydispersity of the suspension tends to shift *ϕ*_m_ towards higher values, as smaller particles can occupy the voids between larger particles. Then, as before, *ϕ*_m_ must be replaced by an effective maximum packing fraction *ϕ*_m,eff_ = *βϕ*_m_ with *β* ≥ 1. Finally, as silica particles may be present in the continuous phase, *η*_c_ must be replaced by *η*_s_ ≥ *η* where *η*_s_ = *ϕ*(*ϕ*_part_) is the viscosity of the suspending phase which may contain silica particles at a volume fraction *ϕ*_part_. It comes:5
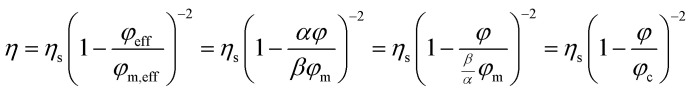
where *ϕ*_c_ = (*β*/*α*) *ϕ*_m_. Since *η* and *ϕ* are known, *η*_s_ and *ϕ*_c_ appear as characteristic parameters specific to each emulsion and are determined by fitting experimental points (see Fig. 6).

In terms of limitation of the model, the literature indicates that other exponents, different of *q* = 2, can be also used. In general, the exponent 2 is the most commonly used exponent.^32,33^ However, other exponents, typically ranging between 1 and 2.5, are also observed in the literature. The exponent of 1 comes from theory of effective media^34^ while the exponents around 2.5 are extracted from Krieger-Dougherty approach.^35^ The factor exponent *q* of 2 stems from an energy minimization approach.^19^ It also appears in dimensional analyses^36^ and experimentally with real hard spheres.^37^

Here, we choose the model with the strongest physical basis and the fewest adjustable parameters. The model chosen here is the simplest and most robust. Furthermore, we do not have many experimental data points. Using more complex models with more adjustable parameters would be incompatible with our limited number of experimental data points. If we want a reasonable number of degrees of freedom, the model should not exceed two adjustable parameters. The two-parameters model is based on energy minimization, and therefore on solid, reliable foundations. So, we use the simplest and most efficient model possible, with sound physical principles. The challenge lies in finding a balance between simplicity, solid physical basis, robustness, and relevant number of degrees of freedom.

Viscosity of the continuous phase (*η*_s_) and the maximal effective fraction (*ϕ*_c_) were fitted to the data through the least square method (eqn (6)). The minimum of the function was calculated thanks to solver tool in Excel®. Algorithm of the macro was a loop that fitted at first *η*_s_, then *ϕ*_c_ and evaluated the difference between previous data (*x*_*n*−1_) and new data (*x*_*n*_) for each parameter. Fitting was accepted by the algorithm when the difference was at least under 0.1% (eqn (7)).6
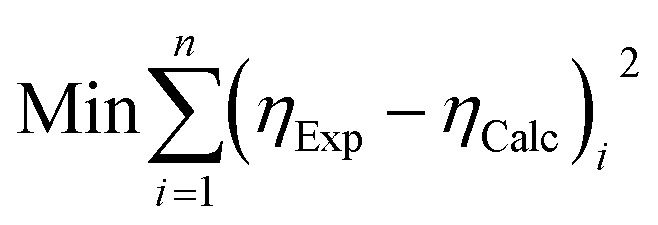
7
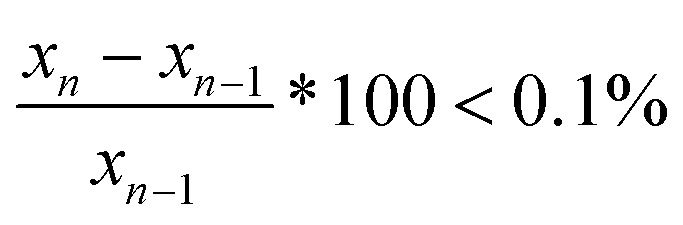


The experimental data agree well with the rheological model (Fig. 6). The obtained fitting parameters are given in Table 2.

**Table 2:** Fitting parameters obtained from the fit of the experimental data of Fig. 6 with eqn (5) (
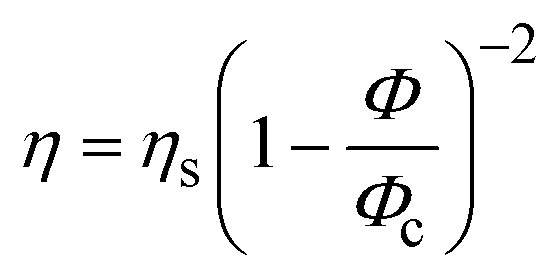
). Viscosity of the continuous phase (*η*_s_) and the maximal effective fraction (*ϕ*_c_) are the two fitting parameters

Process/silica content	*η*_s_ (Pa s)	*ϕ*_c_
Rotor–stator/1 wt%	0.007407	0.79
Sonicator/1 wt%	0.023600	0.74
Rotor–stator/4 wt%	0.006676	0.62

Maximal fractions of dispersed phase predicted by modeling for all the emulsions are not so far from the one experimentally found. It was possible to completely emulsify paraffin oil at a volume fraction of 0.65 but it was not possible to obtain completely emulsification of paraffin oil at a volume fraction of 0.70 (experimentally in our emulsions). This maximal fraction is consistent with those reported with Pickering emulsions where larger dispersed-phase fractions lead to phase inversion or breakage of the emulsions.^6,8,18,28^ Since the maximal fraction (jamming fraction or percolation threshold) corresponds to a solid-like behavior of emulsions, the viscosity at this fraction tends to infinity (asymptotes). This means that dispersed objects into emulsions have very strong and close interactions, like in a solid structure. Formation of a network of droplets is expected. And it means also that these objects are organized in a way that completely fulfills the whole volume, so there is no place to disperse more paraffin oil (or silica particles) into emulsions. The real maximal fraction of dispersed phase (*ϕ*_c_) must be between 0.65 and 0.70 for emulsions in this work. Gaps between the experimental maximal fractions and those predicted by the rheological model have already been observed by other authors and could be attributed to the difficulties in preparing emulsions with high volume fractions of dispersed phase in a robust and repeatable manner.^21,22,38–40^ Experimental error should also play some role on this gap. Furthermore, in every case, viscosities from rheological modeling of the continuous phases (*η*_s_) were higher than experimental viscosity of water (*η*_Water_ = 0.001398 Pa s), predicting the presence of silica particles (so increase of viscosity) in the continuous phases of emulsions.

Looking in details to the fitting parameters (Table 2), we obtained that *ϕ*_c_ is equal to 0.62 for rotor–stator emulsion at 4 wt% of silica, 0.74 for sonicator emulsion at 1 wt% of silica, and 0.79 for rotor–stator emulsion at 1 wt% of silica. At the same time, *η*_s_ reads as 0.006676 Pa s, 0.007407 Pa s, and 0.02360 Pa s for rotor–stator emulsion at 4 wt% of silica, rotor–stator emulsion at 1 wt% of silica, and sonicator emulsion at 1 wt% of silica, respectively. In terms of rheological structural properties analysis, some trends can be extracted based on formulation parameters and processes. As far as the process is concerned, *ϕ*_c_ does not change (*ϕ*_c_ = 0.74 and 0.79) while *η*_s_ is impacted (*η*_s_ = 0.007407 Pa s and 0.02360 Pa s). The *η*_s_ increases when shifting the process from rotor–stator to sonicator. This is due to the network of particles in the continuous phase obtained with the sonicator. Since *ϕ*_c_ does not change, the increase of the prefactor viscosity indicates the mobility of the particles in the network between the droplets. Concerning the effect of formulation parameters, the rheological model highlights that *ϕ*_c_ is affected by the amount of particles at fixed process. The critical *ϕ*_c_ decreases from 0.79 to 0.62 when the particles content increases from 1 wt% to 4 wt%. The jamming or percolation threshold between the droplets occurs at lower dispersed-fraction with the larger amount of silica. The reduction of *ϕ*_c_ proves that the network of silica particles inside the continuous phase creates bridging between the droplets. This confirms the confocal microscopic image of Fig. 4c. This reduction of *ϕ*_c_ was already reported for reverse W/O Pickering emulsions stabilized by silica for which a network of silica was present in the continuous phase.^18^ Conversely, in the presence of BSA proteins only adsorbed at the interface (and not making a network in the continuous phase), *ϕ*_c_ of the order of 0.63–0.64 were obtained.^41^ They correspond well to the *ϕ*_c_ with 1 wt% of silica for which no network of particles can be detected.

For the concentrated emulsions, viscoelastic characterizations can be also used to improve the knowledge of the rheological behavior of the sample. The Fig. S5 of the SIdisplays the elastic G′ and viscous G″ modulus of rotor–stator and Sonicator Pickering emulsions against oscillatory shear strain for emulsions with 60 vol% paraffin oil. Typically, concentrated emulsions are gel-type with a yield stress.

## 4. Conclusions

In this paper, non-conventional anti-Bancroft concentrated Pickering emulsions were studied. The direct oil-in-water emulsions were stabilized by silica particles partially hydrophobized. The paraffin oil dispersed-phase fraction ranges from 0.1 to 0.65. Special attention is paid to the emulsions prepared at the highest dispersed-phase fraction of 0.65. The impact of the process of emulsification (rotor–stator and sonicator shearing devices) and the formulation parameters (1 wt% and 4 wt% of silica particles) on the repartition of the particle and their organization was investigated. Then, the relation between the particle's reparation and the resulting rheological behavior of the emulsions was evaluated.

At a dispersed-phase fraction of 0.65, the droplet size distribution of the rotor–stator emulsions prepared with 1 wt% and 4 wt% of silica as well as the sonicator emulsion prepared at 1 wt% of silica were relatively similar even if smaller population of droplets, of around 10–20 µm, appeared by increasing the silica content to 4 wt% and shifting the stirrer to high energy sonication. The repartition of the particles at the interfaces and in the continuous phase was significantly affected by both the process and the amount of silica. For rotor–stator emulsions at 1 wt% of silica, the large aggregates of particles were mainly anchored on the surface of the droplets. Increasing silica concentration to 4 wt% produced the formation of a dense network of silica in the continuous phase which bridges the droplets. Switching the rotor–stator to sonicator (at 1 wt% of silica) gave rise to emulsion with a network of particles inside the continuous phase between the droplets. The network was less dense than that obtained with rotor–stator emulsion at 4 wt%. All the prepared emulsions are stable over time. This emphasizes that adsorption and/or network of particles in the continuous phase come into play to stabilize the emulsions.

For the rheological properties of the emulsion, the variation the viscosity *versus* the dispersed-phase fraction was followed. A modified version of the model of Quemada 
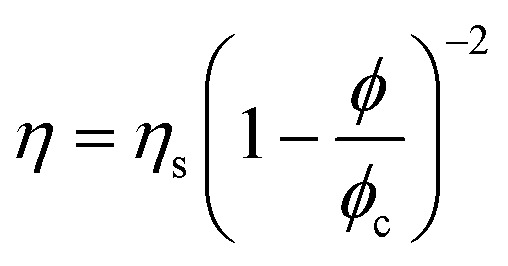
 was used. The model accurately represented the experimental data. The two fitting parameters, *η*_s_ and *ϕ*_c_, were very suitable to represent the repartition of the particles in the emulsions which depended on the stirring process and the amount of silica. The creation of the network of particles inside the continuous phase creating a bridge between the droplets was well represented by the shift of *ϕ*_c_ from 0.79 to 0.62 when the particles content increases from 1 wt% to 4 wt% for rotor–stator emulsions. The apparition of the mobile network of particles in the continuous phase with the sonicator process was accounted by the enhancement of *η*_s_ from rotor–stator emulsions to sonicator emulsions (1 wt% of silica).

The novelty involves in this study concerns mainly the use of the 2 key factors of the Quemada's model to follow the distribution of particles in Pickering emulsions. This is not possible with other rheological models for which a larger number of parameters are used. The 2 parameters of the model produce a rheological signature which is related to the repartition of the particles inside the system. Confocal laser scanning microscopy and mass-balance approaches results confirm and reinforce the organization of the particles deduced from the rheological signature.

## Author contributions

Conceptualization, V. S., P. M., T. H., and T. R. C.; methodology, V. S., D. R., M. A., P. M., and T. R. C.; formal analysis L. B., V. S., D. R., M. A., P. M., and T. R. C.; investigation, V. S., D. R., M. A., P. M., and T. R. C.; resources, V. S., T. H., and T. R. C.; data curation, V. S., D. R., P. M., and T. R. C.; writing—original draft preparation, D. R., V. S., and T. R. C.; writing—review and editing, D. R., V. S., M. A., and T. R. C.; visualization, V. S., D. R., P. M., and T. R. C.; supervision, V. S. and T. R. C.; project administration, V. S. and T. R. C.; funding acquisition, T. H., and T. R. C. All authors have read and agreed to the published version of the manuscript.

## Conflicts of interest

The authors declare no conflicts of interest.

## Supplementary Material

RA-016-D5RA08955G-s001

## Data Availability

All data supporting the findings of this study are available within the article. Supplementary information (SI): five figures showing the droplet size distributions of fresh emulsions and of redispersed creams in silica-free continuous phases, the flow curves of rotor–stator and sonicator emulsions prepared at 1 wt% and 4 wt% of silica for various paraffin oil dispersed-phase contents, and the variations of elastic *G*′ and viscous *G*″ modulus of rotor–stator and sonicator pickering emulsions against oscillatory shear strain (*g*) at different paraffin oil dispersed-phase contents. See DOI: https://doi.org/10.1039/d5ra08955g.
