# Supplementary material for: Effect of formulation parameters and process on the structural properties of concentrated Pickering emulsions
Source: RSC Adv. 2026 Jan 21;16(5):4629–39. doi: 10.1039/d5ra08955g (PMC12821124; doi:10.1039/d5ra08955g)
Supplement: RA-016-D5RA08955G-s001 [file RA-016-D5RA08955G-s001.pdf]

Supplementary Information (SI) for RSC Advances.
This journal is © The Royal Society of Chemistry 2026

Supplementary Information

Effect of formulation parameters and process on the structural properties of concentrated Pickering emulsions

Diego M Ramos [1], Mohammad Mahdi Assaf [1], Véronique Sadtler [1,*], Philippe Marchal [1], Cécile Lemaitre [1], Tayssir Hamieh [2,*], Lazhar Benyahia [3], Thibault Roques-Carmes [1,*]

1 Université de Lorraine, CNRS, LRGP, F-54000 Nancy, France
2 Faculty of Science and Engineering, Maastricht University, P.O. Box 616, 6200 MD Maastricht, The Netherlands;
3 Institut des Molécules et Matériaux du Mans (IMMM), UMR 6283 CNRS – Le Mans Université. 1, Avenue Olivier Messiaen, 72085 Le Mans cedex 9, France
* Correspondence: t.hamieh@maastrichtuniversity.nl, veronique.sadtler@univ-lorraine.fr and thibault.roques-carmes@univ-lorraine.fr

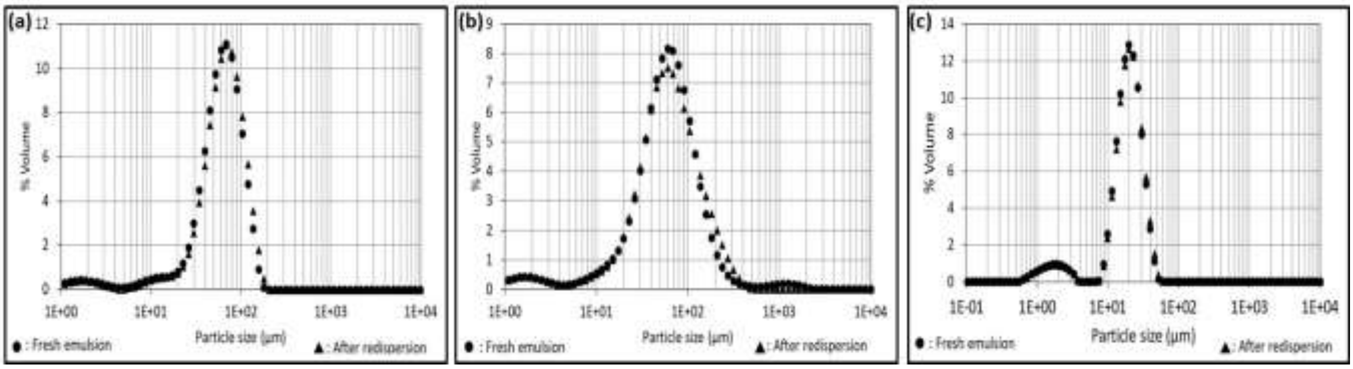

Figure S1. Droplet size distributions of fresh emulsions ("Fresh emulsion") and of redispersed creams in silica-free continuous phases ("After redispersion"). (a,b) Rotor-stator emulsions with 50 vol.% paraffin oil stabilized by (a) 1 wt.% silica particles and (b) 4 wt.% silica particles. (c) Sonicator emulsion with 50 vol.% paraffin oil stabilized with 1 wt.% silica particles.

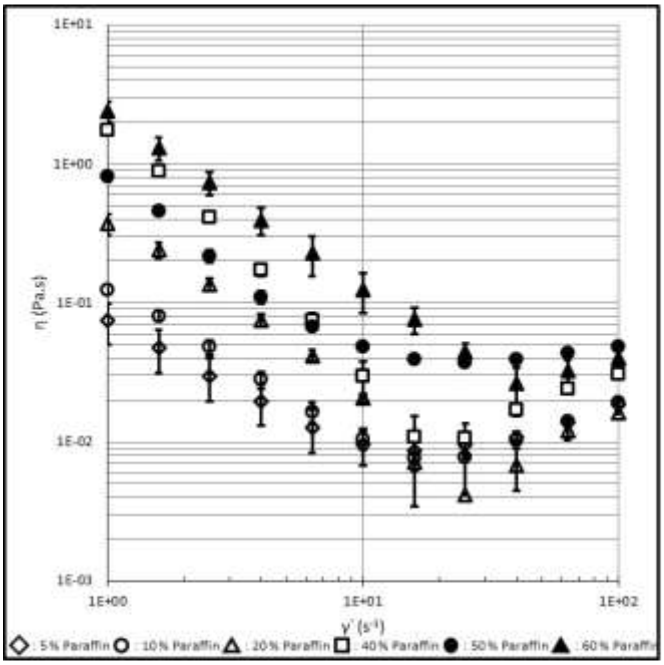

Figure S2. Flow curves of Rotor-stator emulsions prepared at 1 wt.% of silica for various paraffin oil dispersed-phase contents.

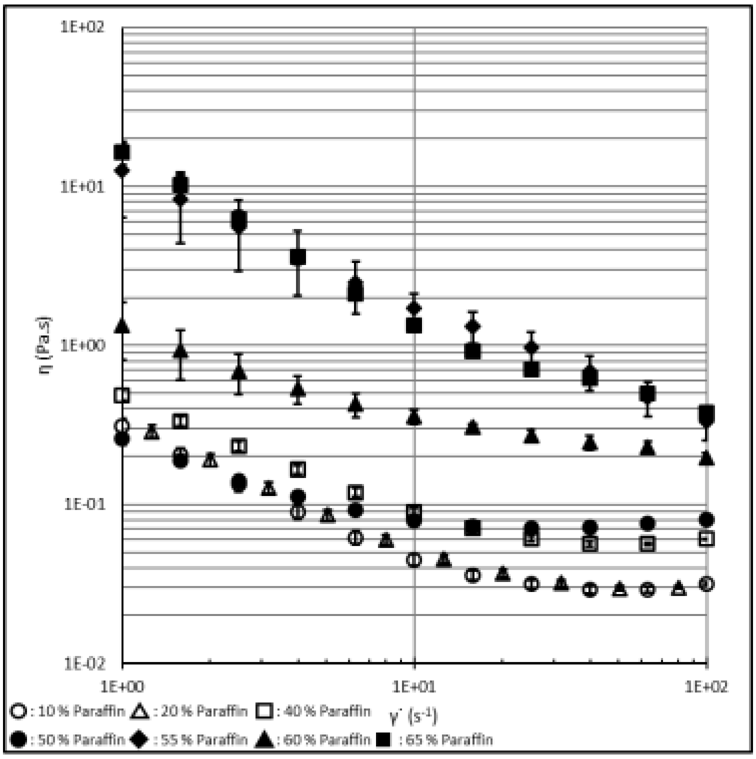

Figure S3. Flow curves of Sonicator emulsions prepared at 1 wt.% of silica for various paraffin oil dispersed-phase contents.

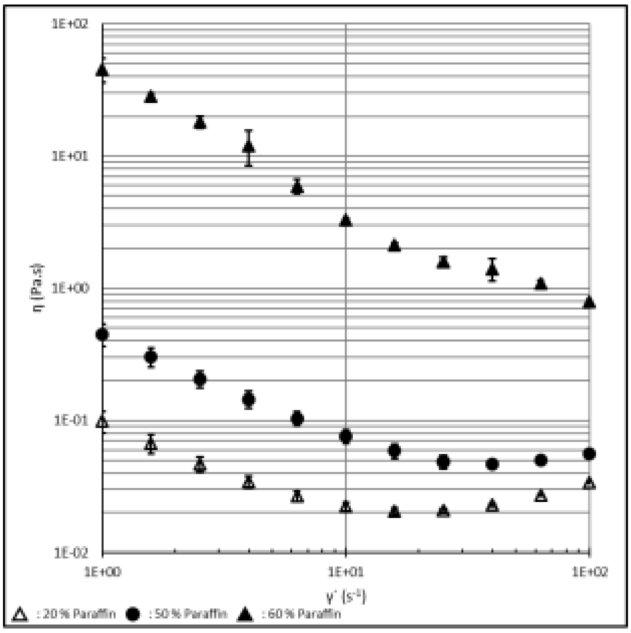

Figure S4. Flow curves of Rotor-stator emulsions prepared at 4 wt.% of silica for various paraffin oil dispersed-phase contents.

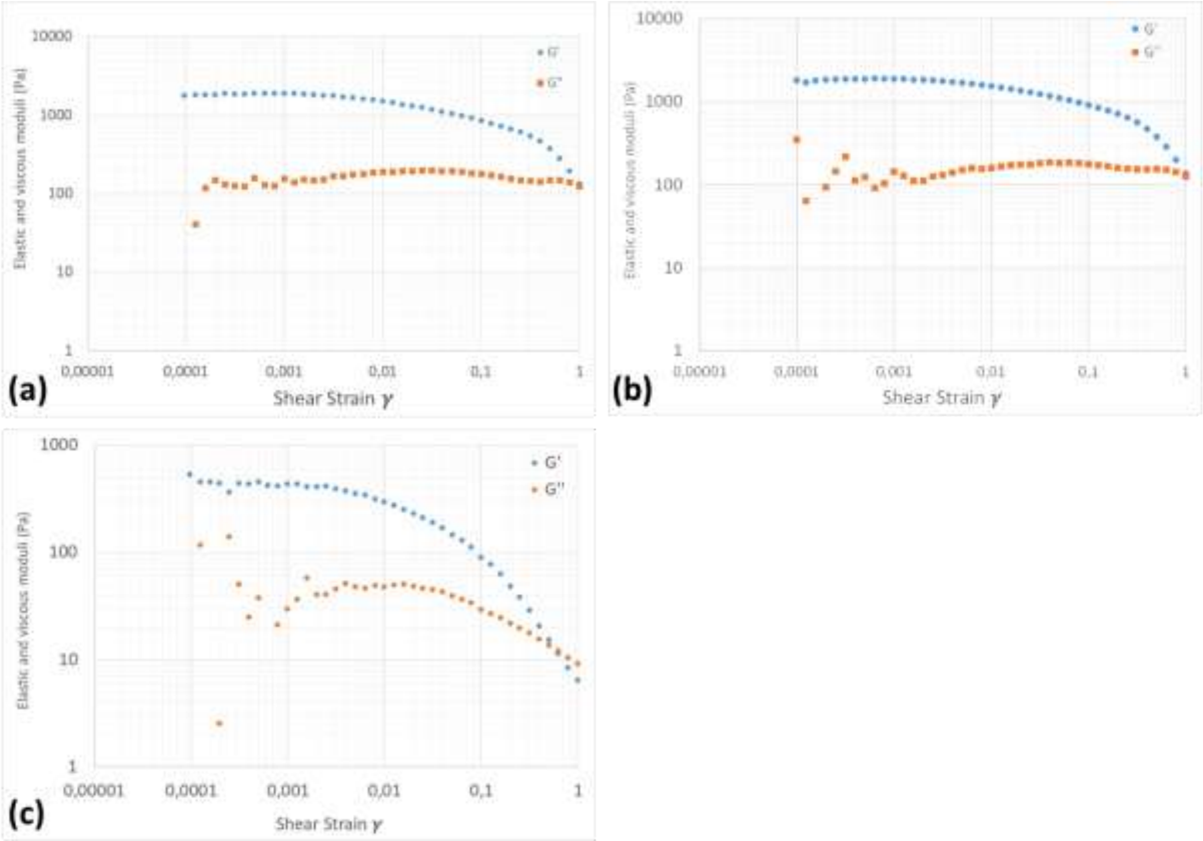

Figure S5. Elastic G’ and viscous G’’ modulus of Rotor-stator and Sonicator Pickering emulsions against oscillatory shear strain (γ). (a) Sonicator emulsion with 60 vol.% paraffin oil stabilized with 1 wt.% silica particles, (b,c) Rotor-stator emulsions with 60 vol.% paraffin oil stabilized by (b) 4 wt.% silica particles and (c) 1 wt.% silica particles.
